# Foreign Body in the Trachea

**Published:** 1851-01

**Authors:** 


*Foreign Body in the Trachea.*—In July last, Mr. J. A. Dobie, of Hanover, N. H., while making an application to his throat by means of a sponge, lost his hold upon it and drew it into his trachea. It could be perceived that the sponge moved from the bifurcation of the bronchi to the larynx. The dyspnœa at times excessive, occasionally disappeared entirely. Tracheotomy was performed by Prof. Dixi Crosby, and the sponge removed. The patient died within forty-eight hours after the operation. The sponge is described as being about two inches long by one wide, and about half an inch in thickness—a size which we should have supposed would entirely preclude its passage through a healthy larynx,—(and such the patient's is said to have been,) or after reaching the trachea, would have prevented it from moving. The fact is interesting, considered in connection with the discussion upon the feasibility of making applications directly to the larynx.—*New Hampshire Jour.*

